# The Present State of Lithium for the Prevention of Dementia Related to Alzheimer’s Dementia in Clinical and Epidemiological Studies: A Critical Review

**DOI:** 10.3390/ijerph18157756

**Published:** 2021-07-22

**Authors:** Nobuyoshi Ishii, Takeshi Terao, Hirofumi Hirakawa

**Affiliations:** Department of Neuropsychiatry, Faculty of Medicine, Oita University, Idaigaoka 1-1, Hasama-machi, Yufu, Oita 879-5593, Japan; nobuy@oita-u.ac.jp (N.I.); hira-hiro@oita-u.ac.jp (H.H.)

**Keywords:** review, lithium, standard levels, trace levels, dementia, dementia prevention

## Abstract

Despite the unavailability of essential anti-dementia drugs, lithium may inhibit glycogen synthase kinase-3 (GSK-3) and decrease beta-amyloid and hyper-phosphorylated tau. In this review, we hypothesized that trace to standard levels of lithium (i.e., corresponding to the therapeutic levels for bipolar disorder) may be effective for dementia prevention. Excluding three insufficient level studies, we obtained two and one excellent clinical studies on standard and trace lithium levels, respectively, all of which supported the effects of lithium for dementia prevention. In addition, we identified good clinical and epidemiological studies (four each) on standard lithium levels, of which six studies supported the effects of lithium. Moreover, of three good epidemiological studies on trace lithium levels, two supported the aforementioned effects of lithium. The number of studies were substantially small, particularly those on trace lithium levels. Moreover, studies on standard lithium levels were insufficient to establish the efficacy of lithium for dementia prevention. This necessitates accumulating good or excellent clinical evidence for the effects of trace to standard lithium levels on dementia prevention.

## 1. Introduction

Lithium therapy is generally accepted as the first-line treatment for bipolar disorder. The standard therapeutic levels range from around 0.4 mEq/L to 1.0 mEq/L whereas such mood-stabilizing effects of lithium disappear less than 0.4 mEq/L in most patients with bipolar disorder. However, the effects of lithium on suicide prevention may be observed from trace to standard levels. A meta-analysis demonstrated an inverse association between trace lithium levels in drinking water and the total and female suicide mortality rates in epidemiological studies [1]. In clinical studies, for example, Kanehisa et al. [2] reported that mean lithium levels in the control group and those in the suicide-attempt group were higher than those in suicide attempters (0.00089 ± 0.00060 mEq/L, 0.00090 ± 0.00046 vs. 0.00068 ± 0.00045 mEq/L) with significant tendency. Multivariate logistic regression analysis with adjustment for age and gender revealed that patients with suicide attempts had significantly lower log-transformed lithium levels than control patients (*p* = 0.032, odds ratio 0.228, 95% CI 0.059–0.883). Moreover, Kurosawa et al. [3] reconfirmed the findings by adjusting for relevant factors, including eicosapentanoic acid and docosahexanoic acid. That is, multivariate logistic regression analysis with adjustment for age, gender, EPA, DHA, arachidonic acid and log-transformed lithium levels revealed that the negative associations with EPA levels (adjusted OR 0.972, 95% CI 0.947–0.997, *p* = 0.031) and log-transformed lithium levels (adjusted OR 0.156, 95% CI 0.038–0.644, *p* = 0.01) and the positive association with DHA levels (adjusted OR 1.026, 95% CI 1.010–1.043, *p* = 0.002) were significant in patients with suicide attempts than in control patients. With regard to standard levels of lithium on suicide, Smith and Cipriani [4] showed evidence for lithium treatment on rates of suicide in patients with mood disorder. Therefore, trace to standard levels of lithium may be effective for suicide prevention.

Dementia is characterized by a deterioration in memory, thinking, behavior, and the quality of life. It develops because of a variety of diseases and injuries that directly and/or indirectly affect the brain. In particular, Alzheimer’s disease (AD) is the most common form of dementia, characterized by the accumulation of beta-amyloid outside neurons and hyper-phosphorylated tau inside neurons. Despite the current unavailability of essential anti-dementia drugs, lithium may decrease beta-amyloid and hyper-phosphorylated tau by inhibiting glycogen synthase kinase-3 (GSK-3) α and β [5,6].

In this review, we hypothesized that trace to standard levels of lithium (i.e., corresponding to the therapeutic levels for bipolar disorder) may be effective for AD prevention.

## 2. Materials and Methods

This review was conducted in May 2021. We searched the PubMed database, augmented by bibliographic cross-referencing to identify articles that investigated the effects of lithium on dementia prevention in humans. The inclusion criteria were as follows: studies had to use the English language, include human subjects, and represent original research data. The exclusion criteria included reviews, commentaries, case reports, and animal studies. We classified the articles according to reports on trace and standard lithium levels which were considered to be so-called therapeutic lithium levels (0.4 to 1.2 mEq/L) or supposed to be exposed to therapeutic lithium prescriptions. They were assessed chronologically and reviewed critically by considering their methods and analyses individually and thereby the level of evidence was rated as excellent, good, or insufficient. “Excellent” was rated for only randomized controlled trial (RCT) and “insufficient” was rated for studies with poor methods or analyses. The others were rated as “good”. This review is not a systematic or meta-analytic review, but a narrative and qualitative review.

## 3. Results

### 3.1. Standard Lithium Levels for Dementia Prevention

Dunn et al. in 2005 [7] performed a case-control epidemiological study in the UK in which 47 and 40 of 9954 dementia cases and 9374 controls, respectively, were exposed to lithium therapy. The lithium dose was not described. The crude odds ratio (OR) for an exposure to lithium was 1.2 (95% confidence interval (CI) 0.8–1.8), which increased to 1.8 (95% CI 1.1–2.8) when adjusted only for the age. However, there was no information on the reason for lithium administration, and if the patients had bipolar disorder or depression. It has been reported that patients suffering from bipolar disorder have a higher risk of developing dementia than those with depression [8]. Therefore, the OR might have reflected bipolar predominance rather than lithium effects because of the inclusion of more patients with bipolar disorder than controls. Dunn et al. suggested that it could possibly be a reverse causation, owing to the use of lithium for depression of dementia. The level of evidence was insufficient.

In contrast, Terao et al. [9] performed a clinical study in Japan in 2006. They compared Mini-Mental State Examination (MMSE) scores between 30 patients receiving lithium and 25 controls and between 35 patients with lithium prescription history (receiving currently and/or in the past) and 20 controls. The mean lithium dose and level was 503 mg/day and 0.66 mEq/L. While the former comparison (mean MMSE score, 27.4 vs. 26.3) was insignificant, the latter (mean MMSE score, 27.5 vs. 25.8) was significant. Moreover, a multiple regression analysis revealed that lithium prescription history significantly predicted MMSE low scores, with an adjustment for the presence of bipolar disorder. The aforementioned findings indicate the effects of lithium on dementia prevention. Nonetheless, the number of patients was extremely small to draw a definite conclusion. The level of evidence was good.

In a clinical study in Brazil, Nunes et al. in 2007 [10] focused on patients with bipolar disorder, and demonstrated that the prevalence of AD was 5% (3 of 66 patients) and 33% (16 of 48 patients) in the group receiving continuous lithium treatment (mean lithium levels were 0.81 mEq/L (SD = 0.18) in normal cognitive function group, 0.78 mEq/L (SD = 0.17) in mild cognitive impairment group, and 0.69 mEq/L (SD = 0.15) in dementia group) and that without lithium treatment but with other mood-stabilizers, respectively. The difference was statistically significant. A multinomial logistic regression analysis revealed an association between lithium and a lower risk of AD (OR = 0.079, 95% CI 0.02–0.32). Terao in 2007 [11] suggested the following two possibilities: (i) lithium might have indirectly prevented dementia via its prophylactic effect on bipolar disorder owing to the increase in dementia rate with relapses [12]; (ii) lithium might have directly prevented dementia via its inhibition of GSK-3. The level of evidence was good.

Angst et al. conducted a clinical study in Switzerland in 2007 [13], and focused on the neurotrophic and neuroprotective properties of lithium, clozapine, and antidepressants. The mean lithium level was 0.7 mEq/L. They investigated the effects of the drugs on dementia prevention in 220 and 186 patients with bipolar disorder and depression, respectively. Multivariable logistic regression analyses revealed an inverse correlation between lithium (OR = 0.36, 95% CI 0.13–1.02) and the severity of dementia in all patients. Moreover, lithium (OR = 0.23, 95% CI 0.06–0.89) and clozapine (OR = 0.11, 95%CI 0.01–0.84) were significantly and inversely correlated with disease severity in 220 patients with bipolar disorder, but not in 186 patients with depression. In addition, mechanisms other than GSK-3, such as neurotrophic and neuroprotective properties, widened the research area for dementia prevention from lithium to clozapine and antidepressants. The level of evidence was good.

In 2008, Macdonald et al. [14] performed an open study where lithium was administered for up to 1 year in the UK, with the lithium side effects rating scale as the primary outcome. Twenty-two patients with AD initiated lithium, of which 14 discontinued the therapy. Patients were commented on low dose lithium carbonate (100 mg/day) and the dosed were titrated for 0.3 and 0.8 mEq/L. Actually, their mean lithium level was 0.4 mEq/L. In contrast, eight patients completed the study. The side effects did not differ between those who discontinued the therapy and the remaining patients. There was no difference in the MMSE scores between the lithium and comparison groups. The study did not report on the effects of lithium on dementia prevention. The primary outcome was side effects, and not effects of lithium on dementia prevention. The level of evidence was insufficient.

In 2008, an epidemiological study conducted in Denmark, Kessing et al. [15], used the data of 16,238 persons who had purchased lithium at least once and 1,487,177 persons who did not purchase lithium. The lithium dose was not described. They conducted a Poisson regression analysis, comprising dementia diagnosis as the outcome and the number of prescriptions for lithium as the variable of interest, with censoring at death or at the end (31 December 2005) of the study period (1995–2005). Three major findings were obtained as follows: (1) persons not exposed to lithium demonstrated a decreased rate of dementia than those who had purchased lithium once (relative risk [RR] = 0.68, 95% CI 0.57–0.82), (2) dementia rate decreased during all periods when people purchased lithium more than once, and (3) dementia rate did not decrease progressively with the number of lithium prescriptions. The first finding might reflect that lithium was predominantly prescribed for bipolar disorder, which increased the risk of developing dementia. The second finding suggests that lithium purchase was associated with a decreased risk of developing dementia, thus indicating lithium effects for dementia prevention. The third finding may deny a dose–response relationship between lithium and dementia. However, the rate of dementia was at similarly low levels among the general population, with little possibility for reduction. Nonetheless, the prescription data for lithium and taking lithium were different, considering the routine problem of non-adherence in psychiatric settings. The level of evidence was good.

In 2009, Hampel et al. [16] performed a randomized placebo-controlled trial for 10 weeks in Ireland to investigate the effects of short-term lithium treatment in patients with AD. A total of 71 patients with mild AD (MMSE scores = 21–26) were randomized to placebo (*n* = 38) or lithium treatment (*n* = 33). The serum lithium levels were titrated to 0.5~0.8 mEq/L. Actually, the mean lithium at the end of treatment was 0.68 mEq/L. Cerebrospinal fluid (CSF) levels of phosphorylated tau (p-tau) and GSK-3 activity in lymphocytes were the primary outcomes. In contrast, CSF concentration of total tau and β-amyloid_1-42_ (Aβ_1-42_), plasma levels of Aβ_1-42_, AD Assessment Scale-Cognitive (ADAS-Cog) summary scores, MMSE, and Neuropsychiatric Inventory were the secondary outcomes. The researchers did not observe any treatment effects on GSK-3 activity or CSF-based biomarker concentrations. Lithium treatment did not change the global cognitive performance, as measured by the ADAS-Cog subscale or depressive symptoms. These results did not support the notion that lithium treatment may reduce hyperphosphorylation of tau protein, following a short 10-week treatment in patients with AD. The method of this study appeared robust. However, the duration of 10 weeks was relatively short to detect lithium effects for dementia prevention. The level of evidence was good.

In 2010, an epidemiological study in Denmark, Kessing et al. [17], focused on the data of 4856 patients with bipolar disorder where 2449, 1781, 4280, and 3901 patients had been exposed to lithium, anticonvulsants, antidepressants, and antipsychotics, respectively, during the study period (1995–2005). The lithium dose was not described. A total of 216 patients were diagnosed suffering from dementia during the follow-up period. Similar to the study by Kessing et al. (2008), the researchers conducted a Poisson regression analysis, comprising dementia diagnosis as the outcome and the number of prescriptions for lithium, anticonvulsants, antidepressants, and antipsychotics as the variables of interest, with censoring during schizophrenia diagnosis, death, or at the end of the study period (31 December 2005). Continued prescriptions of lithium were associated with a decreased rate of dementia ([number of prescriptions]: [2–4], RR = 0.46, 95% CI 0.21–1.01; [5–9], RR = 0.38, 95% CI 0.16–0.86; [10–19], RR = 0.39 95% CI 0.19–0.81; [20~], and RR = 0.44 95% CI 0.23–0.85). Nonetheless, continued prescriptions of anticonvulsants, antidepressants, or antipsychotics were not significantly associated with a decreased rate of dementia. In addition, the prescription data for lithium and taking lithium were different. The level of evidence was good.

In 2011, Forlenza et al. [18] performed a double-blind randomized placebo-controlled 12-month study in Brazil to investigate the effects of long-term lithium treatment on cognitive and biological outcomes in patients with amnestic mild cognitive impairment (aMCI). Forty-five participants with aMCI were randomized to receive lithium (0.25–0.5 mEq/L) (*n* = 24) or placebo (*n* = 21). Lithium treatment for 1 year reduced cognitive decline, compared to placebo. Moreover, long-term lithium treatment was associated with a significant reduction in the CSF concentration of phosphorylated tau. Therefore, lithium may exert a protective effect on the progression of cognitive impairment in dementia. In contrast to the study by Hampel et al., this study had a prolonged (1 year) study period, which may be enough to detect lithium effects for dementia prevention. The level of evidence was excellent.

Gerhard et al. [19] conducted a retrospective, population-based, observational cohort study in the USA. The study included people aged ≥50 years old with bipolar disorder, using combined service and pharmacy claims from 2001 to 2004. Dementia diagnosis was the study outcome. Compared to non-use (0 day), 301–365 days of lithium exposure was associated with significantly reduced dementia risk (hazard ratio [HR] = 0.77, 95% CI 0.60–0.99). However, there was no association for shorter lithium exposures (HR = 1.04, 95% CI 0.83–1.31 for 61–300 days; HR = 1.07, 95% CI 0.67–1.71 for 1–60 days). The lithium dose was not described. In addition, the researchers observed no association for any exposure to anticonvulsants. Therefore, continuous lithium treatment may reduce dementia risk in older adults with bipolar disorder. Similar to the studies by Kessing et al. [15,17], the prescription data for lithium and taking lithium were different. The level of evidence was good.

In 2017, Chen et al. [20] performed a population-based, nested case-control study in Taiwan, using data from the National Health Insurance Research Database from 1997 to 2009. They included a total of 2,548,625 older adults in the study cohort. They analyzed 63,347 cases of AD and two controls per case matched by their age, sex, and index date (the date of the first AD claim). They performed a conditional logistic regression, adjusting for health care utilization, the prescription of valproic acid and carbamazepine, hypothyroidism, type 2 diabetes, hypertension, hyperlipidemia, chronic kidney disease, epilepsy, schizophrenia, and bipolar disorder. They identified 63,347 and 126,694 patients suffering from AD and controls, respectively. The adjusted odds ratio (aOR) of AD risk with lithium use was 1.79 (95% CI, 1.34–2.38) in the general population. However, while restricting the analyses to patients suffering from bipolar disorder to minimize potential confounding factors by indication, lithium administration was not associated with AD risk (aOR = 0.36; 95% CI, 0.89–2.09). Moreover, there were no apparent trends of greater risk with increased duration or dose. The lithium dose was not described. These findings do not support an increased or decreased risk of lithium use with AD, following an adjustment for potential confounding factors. Additionally, in this study, similar to the above studies, prescription data for lithium and taking lithium are different. The level of evidence was good.

In 2019, Forlenza et al. [21] extended their previous study [18] period from 1 year to 2 years, and randomized 61 older adults with MCI to receive lithium (0.25–0.5 mEq/L) or placebo (1:1) (double-blind phase) in Brazil. Moreover, they followed-up the patients for 24 months (single-blinded phase). Cognitive and functional parameters obtained at baseline and after 12 months and 24 months, respectively, were the primary outcome variables. Secondary outcomes included neuropsychological test scores, CSF levels of AD-related biomarkers measured at the time of 0 months, 12 months, and 36 months, and the conversion rate from MCI to dementia (0–48 months). Participants of the placebo group actually showed cognitive and functional decline. In contrast, lithium-treated patients remained stable for over 2 years. The mean lithium levels were 0.39 mEq/L during the double-blind phase, and 0.41 mEq/L during the extension of the trial. Lithium treatment was associated with better performance on memory and attention tests after 24 months, and with a significant increase in CSF amyloid-β peptide (Aβ_1-42_) after 36 months. Therefore, long-term lithium treatment (2 years) attenuated cognitive and functional decline in amnestic MCI, and modified AD-related CSF biomarkers. The level of evidence was excellent.

In 2019, Duthie et al. [22] considered that establishing the dose of lithium that blocks GSK-3 in MCI, a high-risk condition for progression to AD, is an essential step towards a clinical trial for AD prevention using lithium. The lithium levels were 0.33 to 0.54 mEq/L. However, they failed to achieve the desired outcome of establishing a panel of biomarkers for GSK-3 that could be used to track early disease or drug response, and determine the appropriate dose of lithium for inhibiting GSK-3 in MCI volunteers in the UK. This was probably because of the unexpected poor quality of biomarker detection using peripheral blood mononuclear cells. In other words, their study was underpowered. Further sophisticated methods are required to achieve the aforementioned goals. The level of evidence was insufficient.

### 3.2. Trace Lithium Levels for Dementia Prevention

In 2013, Nunes et al. [23] randomized 113 patients with AD having MMSE scores of 9–21 to receive lithium (300 μg/day) (*n* = 58) or placebo (*n* = 55) in a 15-month, randomized, placebo-controlled, double-blind trial in Brazil. The lithium group demonstrated no decline in their performance on the MMSE scores, compared to lower scores in the placebo group. There were significant differences beginning 3 months following the initiation of the treatment, which increased progressively. These findings suggest the efficacy of a microdose lithium treatment in preventing cognitive loss, reinforcing its therapeutic potential in treating AD using extremely low doses. The researchers used a well-designed method. The level of evidence was excellent.

In 2017, Kessing et al. [24] investigated if the incidence of dementia in the general population covaried with long-term exposure to microlevels of lithium in drinking water in Denmark. A total of 73,731 patients with dementia and 733,653 controls (median age, 80.3 years) were included in the study. Lithium exposure was significantly different between patients with dementia diagnosis (median, 11.5 µg/L) and the controls (median, 12.2 µg/L). A nonlinear association was observed in individuals exposed to 2.0 µg/L to 5.0 µg/L lithium. The incidence rate ratio (IRR) of dementia was decreased in those exposed to >15.0 µg/L (IRR = 0.83, 95% CI 0.81–0.85) and 10.1 µg/L to 15.0 µg/L (IRR = 0.98, 95% CI 0.96–1.01) lithium. However, it increased in patients exposed to 5.1 µg/L to 10.0 µg/L lithium (IRR = 1.22, 95% CI 1.19–1.25). Similar patterns were found in those with AD and vascular dementia. Thus, long-term increased lithium exposure in drinking water may be nonlinearly associated with a lower incidence of dementia. However, other unobserved environmental or social care factors related to the municipality of residence might have confounded the association between lithium exposure and dementia rate. This type of epidemiological study may facilitate obtaining indirect evidence. However, similar to lithium prescription, it does not guarantee that the residents actually drank the water and absorbed lithium in their bodies. The level of evidence was good.

In response to Kessing et al. [24], in 2018, Parker et al. [25] used claims data for 4,227,556 adults living in 174 counties in the US. Following county-level demographics (census population, education, Black race, and Hispanic ethnicity) and health care resources (hospital beds per population, primary care physicians per population, psychiatrists per population, and household income), high lithium dose (141.3 µg/L in contrast to 6.0 µg/L of low lithium dose: In detail, there were 142 counties whose lithium levels were equal to or less than 40 µg/L and 32 counties whose lithium levels were more than 40 µg/L. The authors’ high lithium dose was the mean of lithium levels of the 32 counties while low lithium dose was that of the 142 counties.) did not confer any significant benefit for bipolar disorder, dementia, or major depressive disorder. Despite the association being sufficiently adjusted, the evidence was indirect. The level of evidence was good.

In 2018, Fajardo et al. [26] conducted an epidemiological study to examine the relationship between trace levels of lithium in drinking water and changes in AD mortality across several Texas counties. They hypothesized a negative association between trace levels of lithium and changes in AD mortality. This can be attributed to the steady increase in AD mortality rates throughout the years. To calculate the change in AD mortality, they obtained the age-adjusted AD mortality rates (per 100,000) for each county between 2000 and 2006 and between 2009 and 2015. Moreover, the change in AD mortality was calculated by subtracting the rate obtained between 2000 and 2006 from those obtained between 2009 and 2015. Consistent with their hypothesis, trace lithium levels in water were negatively associated with changes in AD mortality. The mean lithium dose was 56 µg/L. In addition, counties with less than the median level of 40 µg/L lithium in water experienced significantly greater increases in age-adjusted AD mortality, compared to those with lithium above the median level. However, the study does not guarantee that the residents actually drank water and absorbed lithium in their bodies. The level of evidence was good.

## 4. Discussion

As shown in Table 1, we identified 13 studies on standard lithium levels for dementia prevention, namely five epidemiological studies and eight clinical studies. In contrast, we identified four studies on trace lithium levels for dementia prevention, namely three epidemiological studies and one clinical study. Excluding three studies with insufficient level of evidence, we obtained two and one excellent clinical studies on standard and trace lithium levels, respectively, all of which supported the effects of lithium for dementia prevention. In addition, we obtained good clinical and epidemiological studies (four each) on standard lithium levels, of which six studies supported the effects of lithium for dementia prevention. Of three good epidemiological studies on trace lithium levels, two supported the effects of lithium for dementia prevention. However, the number of studies is currently extremely small, particularly those on trace lithium levels. Moreover, there are inadequate studies on standard lithium levels to conclude if lithium is effective for dementia prevention.

If lithium prevents dementia, lithium might have indirect and direct dementia prevention effects. Since many studies demonstrated that depressive symptoms are a risk factor of dementia, the indirect dementia prevention effect might be large, although it is unknown whether the indirect effect is larger or smaller than the direct effect of lithium on dementia prevention in subjects with bipolar disorder or depression. On the other hand, the three previous studies rated as excellent suggested the direct dementia prevention effect in mild cognitive impairment and Alzheimer’s disease. Further studies are required to investigate the indirect and direct dementia prevention effects of lithium.

Despite the importance of epidemiological studies on lithium prescription and its levels in drinking water, they are inconclusive because of indirect evidence. Although pre-clinical studies (in vitro and with animal models) were not collected in this review, such pre-clinical studies may provide some positive evidences for supporting the idea that more clinical trials should be done with lithium treatment (or lithium in water). Additionally, this necessitates further clinical studies to confirm the aforementioned findings. For example, in the association between suicide rate and lithium in drinking water, we performed clinical studies [2,3] to directly confirm the indirect evidence from epidemiological studies [27,28,29,30] although we could not perform RCTs for that field.

Limitations of the present review are: (1) the method of searching relevant references are not systematic, but arbitrary, (2) although we tried to rate individual studies as excellent, good, and insufficient in the viewpoint of their methods and analyses with reasons for the rating, there was no consistently objective and robust way to rate the studies, and (3) the number of studies cannot be essentially used as a parameter of evidence levels.

Nonetheless, we believe that the present review is worthy because it critically pointed out the lack of sufficient evidence for lithium effects on dementia prevention and warranted further clinical studies.

## 5. Conclusions

At present, the efficacy of lithium for dementia prevention is unclear. Therefore, it is necessary to accumulate good or excellent clinical evidence on the effects of trace to standard lithium levels for dementia prevention.

## Figures and Tables

**Table 1:** Number of epidemiological or clinical studies of standard lithium levels or trace levels on dementia prevention.

	Studies on Standard Lithium Levels		Studies on Trace Lithium Levels	
Evidence levels	Number of epidemiological studies	Number of Clinical studies	Number of epidemiological studies	Number of clinical studies
Excellent	0	2 (2)	0	1 (1)
Good	4 (3)	4 (3)	3 (2)	0
Insufficient	1 (0)	2 (0)	0	0

The number of studies supporting the effects of lithium for dementia prevention is in parenthesis.

## Data Availability

No data were generated during this study.
